# Evidence for SARS-CoV-2 infected Golden Syrian hamsters (*Mesocricetus auratus*) reducing daily energy expenditure and body core temperature

**DOI:** 10.1038/s41598-024-73765-2

**Published:** 2024-10-06

**Authors:** Björn-Patrick Mohl, Claudia Blaurock, Alexander Riek, Catherine Hambly, John R. Speakman, Anne Balkema-Buschmann

**Affiliations:** 1 Institute of Novel and Emerging Infectious Diseases, Friedrich-Loeffler-Institut, Greifswald-Insel Riems, Germany; 2 Institute of Animal Welfare and Animal Husbandry, Friedrich-Loeffler-Institut, Celle, Germany; 3 School of Biological Sciences, University of Aberdeen, Aberdeen, UK

**Keywords:** Microbiology, Virology, SARS-CoV-2

## Abstract

Golden Syrian hamsters (*Mesocricetus auratus*) are a well-established animal model for human infections with severe acute respiratory syndrome coronavirus 2 (SARS-CoV-2) due to their susceptibility to SARS-CoV-2 infection, robust virus replication and pathological manifestations similar to human COVID-19 pneumonia. To investigate the physiological changes upon infection in this animal model, we explored the alterations in daily energy expenditure (DEE), water turnover, body mass, body temperature, and locomotor activity in non-infected and SARS-CoV-2 infected Golden Syrian hamsters for four days post SARS-CoV-2. DEE was measured using the doubly labelled water method, which allows for the accurate estimation of carbon dioxide production and, consequently, energy expenditure in animals. Additionally, we investigated total water intake (TWI), which comprises drinking water, preformed water in food, and metabolic water. Using intraperitoneally implanted data loggers, we also monitored body core temperature and locomotor activity in some of the animals. Here we provide evidence for infected hamsters exhibiting significantly lower DEE and TWI compared to non-infected animals. We also observed an increase in body weight in the non-infected animals, while infected animals experienced weight loss. Further, infected animals showed a significantly decreased body temperature, indicating a generally lowered metabolic rate.

**Supplementary Information:**

The online version contains supplementary material available at 10.1038/s41598-024-73765-2.

## Introduction

The outbreak of the severe acute respiratory syndrome coronavirus 2 (SARS-CoV-2) in late 2019 has resulted in a global pandemic, with significant implications for public health and the global economy. The Golden Syrian Hamster (*Mesocricetus auratus*) is one of the most reliable COVID-19 animal models, as it mimics the disease progression and pathology in humans. Previous studies have demonstrated that viral infections can induce alterations in energy expenditure and body mass in humans^1^. These alterations are often attributed to factors such as loss of appetite, reduced food consumption, increased cytokine release, increased glucose metabolism and fatigue-induced reduction in physical activity^2,3^. Moreover, studies investigating SARS-CoV-2 infection in humans have reported weight loss and decreased muscle mass^4,5^, further emphasizing the impact of viral infections on energy metabolism.

However, there is still a significant knowledge gap regarding the specific metabolic changes associated with SARS-CoV-2 infection and their underlying mechanisms. Furthermore, studies focusing on the alterations in total water intake (TWI) during viral infections, including SARS-CoV-2, are lacking. Understanding the changes in water metabolism during viral infections can provide valuable insights into the broader physiological consequences and potential therapeutic targets.

Using the Golden Syrian Hamster model, we analysed DEE, body mass and TWI within the first four days after infection, as well as body core temperature (and locomotor activity in a single hamster per group) or seven days after infection. This allowed us an insight into the metabolic state of non-infected and infected animals. This will improve our understanding of the metabolic and physiological changes associated with SARS-CoV-2 infection in humans and potentially identify novel therapeutic targets to mitigate the metabolic consequences of the infection, while providing additional monitoring criteria associated with this disease.

## Results

### Significantly reduced daily energy expenditure (DEE) and total water intake (TWI) in infected Golden Syrian Hamsters versus non-infected hamsters

**Fig. 1:**
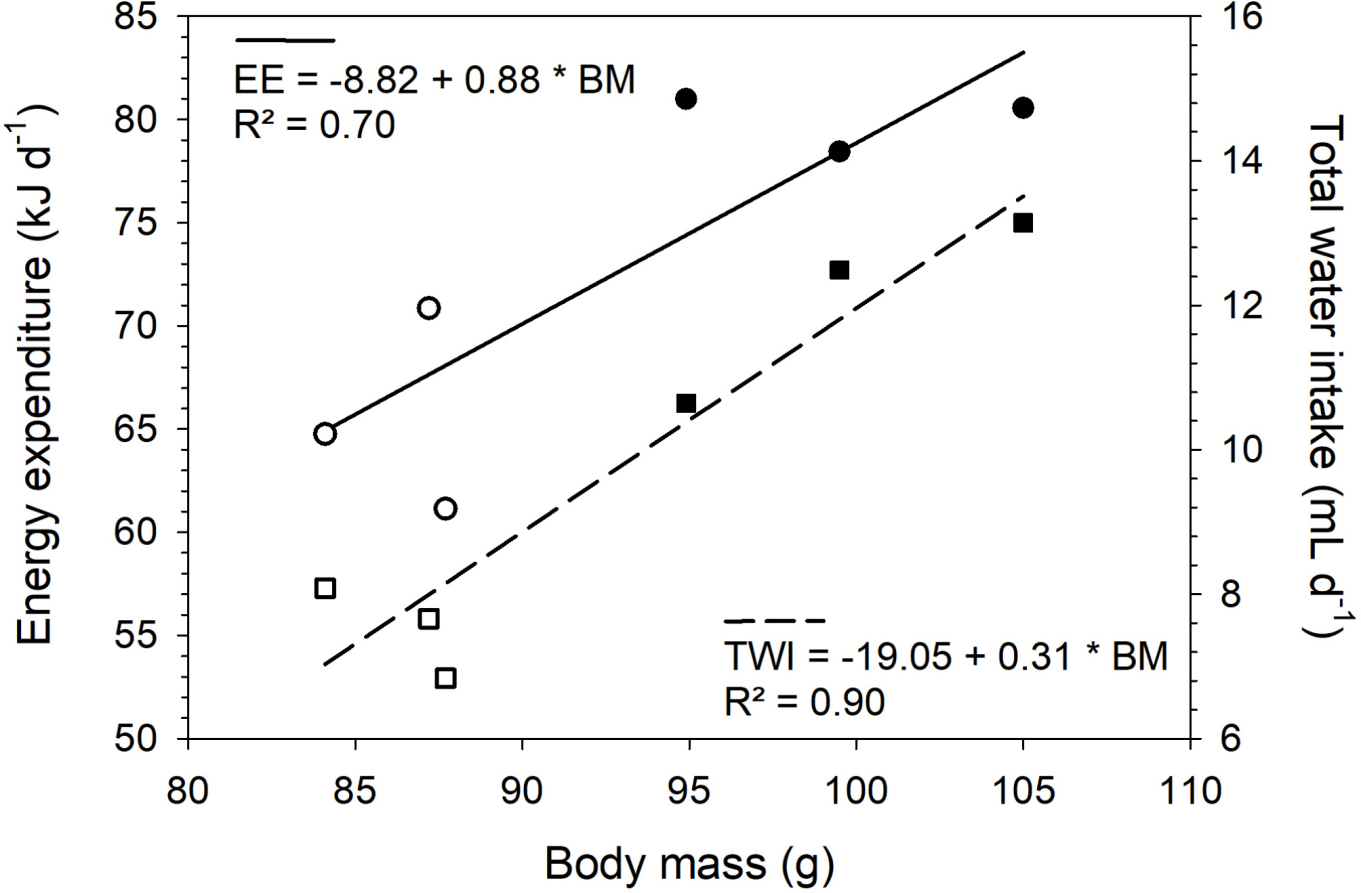
Relationship between daily energy expenditure (circles, DEE), total water turnover intake (squares, TWI) and body mass (BM) measured using the doubly labelled water method in non-infected (closed symbols) and SARS-CoV-2 infected (open symbols) Golden Syrian Hamsters (*Mesocricetus auratus*).

After inoculation with 1*10^2^ TCID_50_ SARS-CoV-2, SARS-CoV-2 infected hamsters exhibited significantly (*P* = 0.029) lower DEE (65.6 ± 2.8 kJ/d, [± SE]) compared to non-infected animals (80.0 ± 0.8 kJ/d), with the lowest recorded DEE in an infected animal being 61.14 kJ/d and the highest in a non-infected animal being 81.0 kJ/d (Table 1; Fig. 1). Similar to DEE, TWI differed significantly (*P* = 0.013) between both groups, with non-infected animals having a higher water intake (12.1 ± 0.8 mL/d) compared to SARS-CoV-2 infected animals (7.53 ± 0.4 mL/d; Table 1; Fig. 1, Suppl. Table 1).

**Table 1:** Body mass (BM), dilution spaces for ^18^o (N_O_) and^2^H (N_H_), respective turnover rates (k_O_, k_H_), total water intake and daily energy expenditure in non-infected and SARS-CoV-2 infected Syrian hamsters over a measuring period of four days (means ± SE).

Parameters		Non-infected	SARS-CoV-2 infected^1^	Welch T-test	
		(*n* = 3)	(*n* = 3)	t	P-value
Initial BM	(g)	94.7 ± 2.7	93.0 ± 1.4	0.56	0.612
Final BM	(g)	99.8 ± 2.9	86.3 ± 1.1	**4.31**	**0.031**
BM change	(g)	5.1 ± 0.3	− 6.6 ± 0.6	**16.98**	**< 0.001**
N_O_	% of BM	63.2 ± 1.7	63.1 ± 1.0	0.06	0.956
N_H_	% of BM	65.3 ± 1.7	65.2 ± 1.0	0.05	0.964
k_O_	(d^− 1^)	0.29 ± 0.01	0.22 ± 0.01	**4.94**	**0.009**
k_H_	(d^− 1^)	0.19 ± 0.01	0.13 ± 0.01	**4.69**	**0.017**
Total water intake	(mL d^− 1^)	12.1 ± 0.8	7.53 ± 0.4	**5.48**	**0.013**
(mL kg^− 0.83^ BM d^− 1^)		83.5 ± 3.4	55.8 ± 3.2	**5.93**	**0.004**
Energy expenditure	(kJ d^− 1^)	80.0 ± 0.8	65.6 ± 2.8	**4.90**	**0.029**
(kJ g^− 0.75^ BM d^− 1^)		2.6 ± 0.1	2.3 ± 0.1	**2.96**	**0.047**

Statistically significant differences are printed in bold.

^1^Animals were infected with SARS-CoV-2 the same day the doubly labelled water measurements started.

### SARS-CoV-2 infection result in reduced body mass in infected hamsters

In addition to DEE, our study also observed significant changes in body mass over the four-day measurement period between infected and non-infected animals. At the start of the study, body mass did not differ between both groups. However, infected animals were found to lose weight during the time of DEE measurements, while non-infected animals gained weight. The study revealed significant (*P* < 0.001) changes in body mass over the four day measurement period with non-infected animals increasing their body mass (5.1 ± 0.3 g) while infected animals experienced weight loss (− 6.6 ± 0.6 g) (Table 1, Suppl. Table 1).

### Decreased body temperature observed in infected hamsters.

**Fig. 2:**
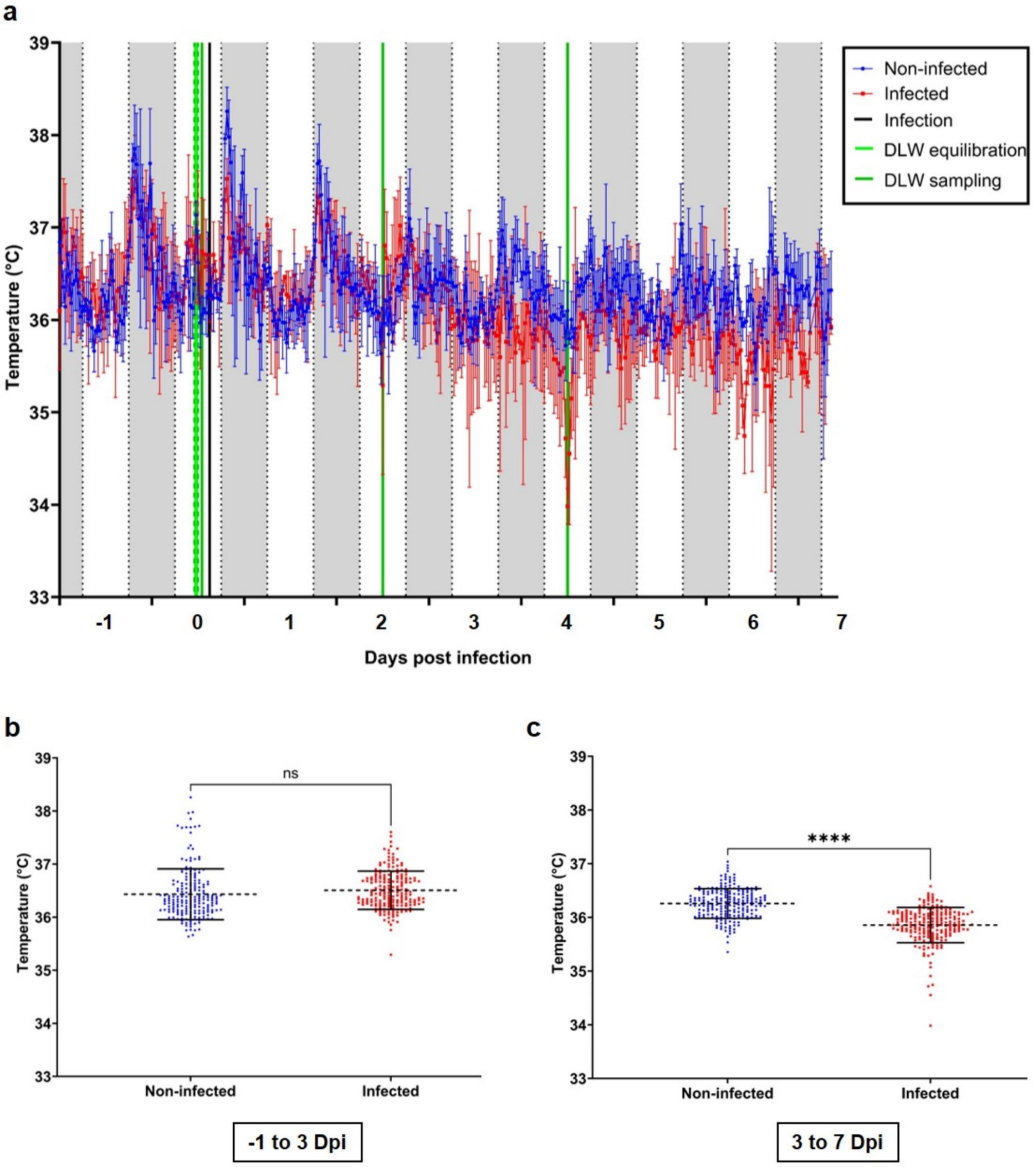
Body core temperature of Golden Syrian Hamsters infected with 10^2^ TCID_50_ SARS-CoV-2. Non-infected (blue) and infected (red) hamsters were monitored for body core temperature changes and the 30 min averages were calculated and displayed from − 1 until 7 days post infection (dpi). (**a**) Body temperature of non-infected and infected hamsters, error bars indicate standard deviation. Daytime (6 am to 6 pm) is marked in white, nighttime (6 pm to 6 am) is marked in grey. The infection time-point is depicted by a black line. Doubly labelled water injection/equilibration (light green) and sample collection (dark green) are indicated. (**b**) Body temperature distributions between − 1 and 3 dpi, mean is indicated by dotted-line, error bars indicate standard deviation. (**c**) Body temperature distributions between 3 and 7 dpi, mean is indicated by dotted-line and the error bars indicate standard deviation. Statistical analysis used was an unpaired t-test (two-tailed), significant differences are denoted by asterisks (*p* < 0.0001), and non-significant by ns (*n* = 3).

**Fig. 3:**
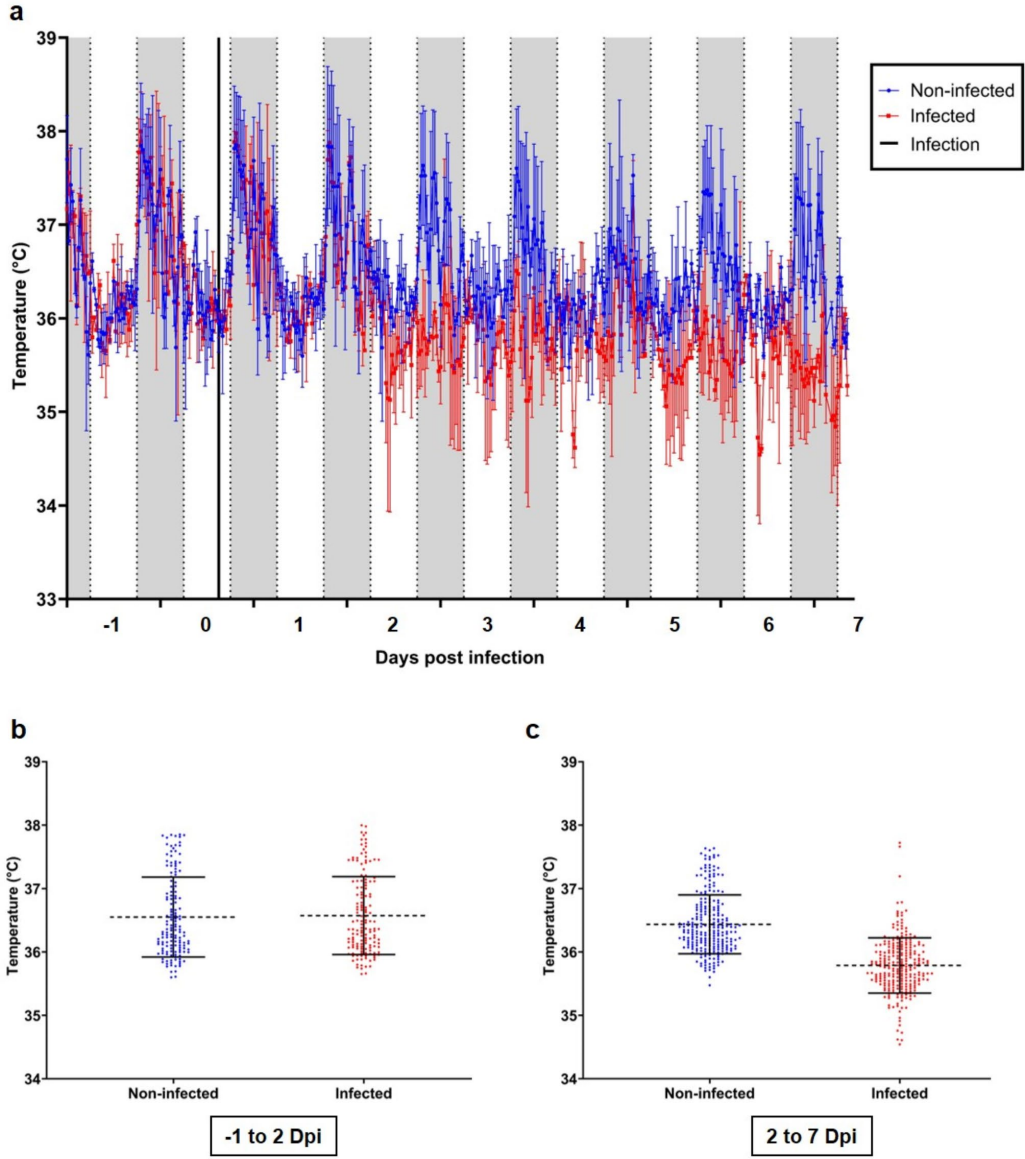
Body core temperature of Golden Syrian Hamsters infected with 10^4^ TCID_50_ SARS-CoV-2. Non-infected (blue) and infected (red) hamsters were monitored for body core temperature changes and the 30 min averages were calculated from − 1 until 7 days post infection. (**a**) Body temperature of non-infected and infected hamsters, error bars indicate standard deviation. Daytime (6 am to 6 pm) is marked in white, nighttime (6 pm to 6 am) is marked in grey. The infection time-point is depicted by a black line. (**b**) Body temperature distributions between − 1 and 2 dpi, mean is indicated by dotted-line and error bars indicate standard deviation. (**c**) Body temperature distributions between 2 and 7 dpi, mean is indicated by dotted-line and the error bars indicate standard deviation (*n* = 2).

Following challenge with 1*10^2^ TCID_50_ SARS-CoV-2, the body temperature of infected hamsters decreased, beginning to diverge from the non-infected control animals at three days post infection, and remaining lower until the conclusion of the experiment at seven days post infection (Fig. 2a). Between minus one and three days post infection, no significant difference (*p* = 0.0798) in body temperature was determined between non-infected and infected hamsters (Fig. 2b, Suppl. Table 1). However, between three and seven days post infection, the body temperature of infected hamsters was significantly (*p* < 0001) lower compared to the non-infected hamsters (Fig. 2c, Suppl. Table 1). Moreover, in a follow-up study, hamsters challenged with 1*10^4^ TCID_50_ SARS-CoV-2 displayed a similar decrease in body temperature relative to the non-infected control. In this study, the infected hamsters’ body temperature decreased and diverged from the non-infected control animals from two days post infection until the conclusion of the experiment at seven days post infection (Fig. 3a). Here, we observed a body temperature parity between non-infected and infected hamsters until two days post infection (Fig. 3b), and could note a meaningfully lower body temperature in infected hamsters relative to the non-infected controls between two- and seven-days post infection (Fig. 3c).

**Fig. 4:**
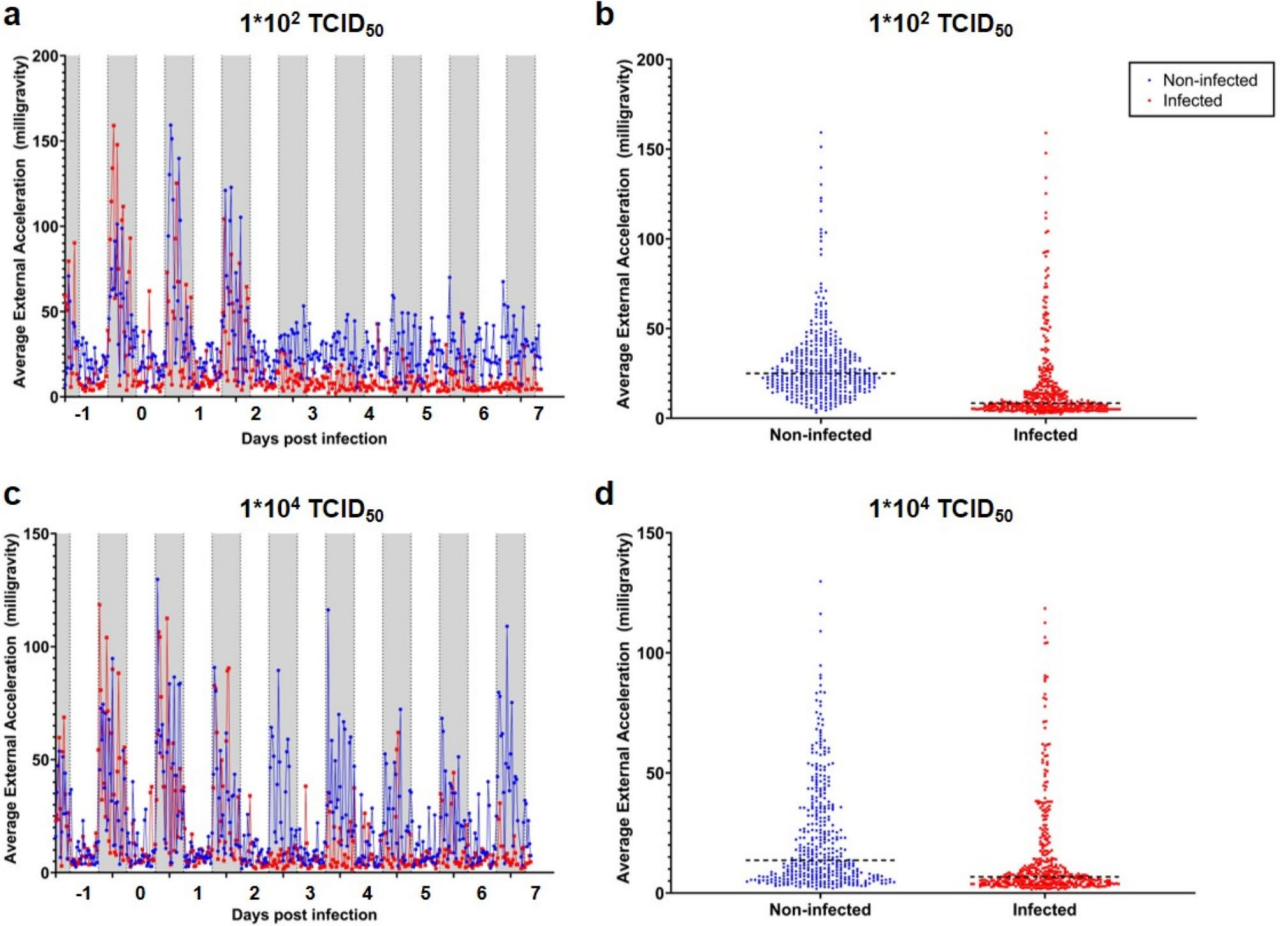
Locomotor activity of non-infected and SARS-CoV-2 infected Golden Syrian Hamster. One non-infected (blue) and one infected (red) hamster were monitored for locomotor activity (average external acceleration in milligravity units), 30 min averages are displayed from − 1 until 7 dpi. (**a** and **b**) A non-infected and a 10^2^ TCID_50_ SARS-CoV-2 infected hamster, (**c** and **d**) a non-infected and a 10^4^ TCID_50_ SARS-CoV-2 infected hamster. (**a** and **c**) Average external acceleration during daytime (6 am to 6 pm; white) and nighttime (6 pm to 6 am; grey). (**b** and **d**) Average external acceleration measurement distributions between − 1 and 7 dpi, the dotted line indicates the median of the data (*n* = 1).

Interestingly, as one of the utilized data loggers also monitored locomotor activity, we also have the average external acceleration data for one non-infected and one infected hamster corresponding to the body temperature data presented in Figs. 2 and 3 for both infection doses. Here we could observe a decrease in locomotor activity by two days post infection until seven days post infection for the hamster infected with 1*10^2^ TCID_50_ (Fig. 4a and b) and 1*10^4^ TCID_50_ (Fig. 4c and d) SARS-CoV-2. However, as these data only represent single animals, we cannot ascribe any significance to these observations. Furthermore, the Shapiro-Wilk test was employed to assess the normality of the data dividing to individuals classified as “infected” and “non-infected”. Since the significance value from the Shapiro-Wilk is greater than 0.05, we can conclude that data follows a normally distribution.

## Discussion

We observed a general metabolic downregulation in the infected animals, as determined by the parameters of DEE, TWI, and body core temperature. One explanation for the lowered DEE and body mass could be the loss of appetite and the resulting reduced food intake, leading to a loss in body weight as observed in our study. In fact, besides the typical clinical signs of a SARS-CoV-2 infection in hamsters such as decreased general condition and respiratory symptoms, we did observe a reduced feed intake in the infected animals, which developed at 3–4 days post infection and lasted until 6–7 days post infection. This finding is consistent with previous studies that have reported weight loss, changes in body composition and decreased muscle mass during SARS-CoV-2 infections in humans^4–6^ and is defined as an important parameter in SARS-CoV-2 infected hamsters at this timepoint^7,8^. Furthermore, many viral infections cause symptoms of fatigue and lethargy that are triggered by immune-to-brain signalling pathways during the inflammatory response^9^ and might result from the body’s attempt to conserve energy by reducing activity. Viral infections generally trigger the release of cytokines, which are signalling molecules involved in immune response and inflammation. Excessive cytokine release, known as a cytokine storm, can occur during severe viral infections including SARS-CoV-2 and lead to a wide range of symptoms, including fatigue and decreased energy expenditure^2,3^. One possible reason for the decreased water intake in infected animals could be a decreased renal function, as has been reported for COVID-19 patients^10^.

A decrease in body temperature in influenza virus infected mice has been described earlier^11^, and the authors postulated that this could be a result of the decreased food intake in combination with cytokine induced reaction to the virus infection. In fact, Golden Syrian Hamsters are capable of hibernation^12,13^, and thus are well prepared to low body temperatures and do not suffer from metabolic alterations correlated to a body temperature below 35 °C. Therefore, although the expected reaction to a virus infection would have been the development of fever in most mammalian species, the inflammation-induced downregulation of the body temperature in SARS-CoV-2 infected hamsters measured here using two different infection doses may also indicate their mechanism of defence against the virus infection. In human COVID-19 patients, hypothermia is observed in patients with an increased mortality rate^14^. However, hypothermia and hibernation have distinct causal features^15^, and it may be conjectured that in response to the virus infection, the hamsters enter a pre-hibernation state, which entails decreases in body temperature and body mass^16^.

In conclusion, our study provides evidence for SARS-CoV-2 infected Golden Syrian Hamsters exhibiting a significant decrease in DEE and TWI compared to non-infected animals, which is associated with changes in body mass. Monitoring physiological parameters in individual animals using data loggers confirmed a reduction in body core temperature in infected animals, supporting the assumption that a SARS-CoV-2 infection results in a general metabolic downregulation in this species. A notable limitation of our study is the small sample size, with only three samples in each group. This limited sample size restricts the statistical power of our analysis and somewhat compromises the extension of our findings to larger populations. A small sample size increases the risk of type I and type II (specially type II) errors, potentially leading to overestimation (underestimation) of the true effect sizes. Also, the small sample size may limit the exploration of a potential variable that influences other variables and subgroup analyses, thus placing a statistical constraint on the depth of insights derived from the study. While we have tried to employ accurate statistical methods to reduce these limitations, including effect size estimation and sensitivity analyses, the inherent risk variability within small sample sizes warrants cautious interpretation of the results. Thus, further research is warranted to better understand the mechanisms underlying these observations and their implications for energy homeostasis and fluid balance in the context of SARS-CoV-2 infections in hamsters as animal models for human disease, and to consider parameters such as body temperature and possibly also locomotor activity when assessing physiological changes.

## Methods

### Ethics statement

Hamster experiments were carried out according to the German Regulations for Animal Welfare after obtaining the necessary approval from the authorized ethics committee of the State Office of Agriculture, Food Safety and Fishery in Mecklenburg – Western Pomerania (LALLF MV) under permission number 7221.3-1-049/20 and approval of the commissioner for animal welfare at the Friedrich-Loeffler-Institute (FLI), representing the Institutional Animal Care and Use Committee (IACUC). The study is reported in accordance with ARRIVE guidelines.

## Animals

Male Golden Syrian Hamsters (*Mesocricetus auratus*; RjHan: AURA) that were five to seven weeks old with a body weight of 80–100 g were obtained from Janvier Labs (Saint Berthevin, France). The experiments were conducted in a BSL-3 animal facility. The present study was part of a larger study on SARS-CoV-2 in hamsters^17^, and the DEE data has been summarized there. Hamsters were housed in groups of three in individually ventilated cages. Three of these hamsters were inoculated by the orotracheal route with 1*10^2^ TCID_50_, and in a separate study, another group of three hamsters were orotracheally inoculated with 1*10^4^ TCID_50_ SARS-CoV-2 Germany/BavPat1/2020 (BavPat1)^18^ (GISAID accession EPI_ISL_406862) in a volume of 100 µL, while the others remained non-infected. Animals had ad libitum access to pelleted food (ssniff, Soest, Germany) and water. Cages were filled with Abedd Aspen CLASSIC bedding (ssniff, Soest, Germany) and hay. The animals’ well-being and body weight were checked daily. Animals were clinically observed for 7 days with a daily sampling for virological analysis. After 7 days, animals were euthanized by inhalation of an isoflurane overdose followed by intracardial exsanguination and decapitation.

### Daily energy expenditure and water turnover

The daily energy expenditure (DEE) was determined individually for six hamsters (three non-infected and three hamsters infected with 1*10^2^ TCID_50_ SARS-CoV-2) for a total of four days using the doubly labelled water (DLW) method^19,20^. The method and sample analysis are explained in detail elsewhere^21,22^. In brief, before dosing a blood sample was taken of every animal to estimate the background isotopic enrichment of ^2^H and^18^O. Hamsters were then injected intraperitoneally with 1.99 ± 0.03 g DLW per kg body mass, (65% 18O and 35% 2H; 99.90% purity). The individual dose for each hamster was determined prior to the injection according to its body mass. Subsequently, after a one-hour equilibration period, blood samples of 70–100 µL were drawn by punctuation of the gingival venous plexus of every hamster at 1, 48 and 96 h after injection to estimate the isotope elimination rates. Serum samples were stored at − 20 °C until determination of 18O and 2H enrichment. For analysis of the samples for 18O and 2H see references above. The DEE was calculated from carbon dioxide production by assuming a respiration quotient of 0.85. Isotope analyses and calculations were made blind of the status of the animals. The total water intake (TWI; mL/d) that consists of drinking water, preformed water ingested in food and metabolic water was estimated as the product of the deuterium space and the deuterium turnover rate^23^. Groups were compared using a Welch T-test.

## Core body temperature and locomotor activity monitoring

One week before the study, data loggers (either a Nano-T, Micro-HRT, or DST micro-ACT (Star Oddi, Gardabaer, Iceland)) were implanted intraperitoneally. One hour pre-operation, animals were injected subcutaneously (s.c.) with 0.2 mg/kg Meloxicam (Metacam, Boehringer Ingelheim Vetmedica, Germany) for analgesia. Under isoflurane inhalation, a 1 cm incision was placed in the linea alba and the data logger was carefully inserted into the abdomen without fixation to the muscular layer of the abdomen wall. During surgery, 5 mg/kg of Enrofloxacin (Baytril, Bayer Leverkusen, Germany) were instilled intraperitoneally as an antibiotic. The muscular layer as well as the outer skin were closed separately using non-absorbable nylon (USP 4/0; SMI AG St. Vith, Belgium). The wounds were checked daily until the end of the experiment. All data loggers were programmed to record temperature (°C), and one set of DST micro-ACT data loggers, but not the Nano-T or Micro-HRT data loggers, recorded acceleration-based activity every 10 min throughout the study. The DST micro-ACT logger measured the acceleration in three axes using a defined sampling frequency for one minute, then the algorithm calculated the minimum, maximum, and average external acceleration (EA) and the variance of these variables. The EA is the acceleration above standard gravity defined with a calibration and calculated as the vectoral sum of body acceleration (VeDBA) in milli-gravity units. During the necropsy, the data loggers were recovered, and the data was extracted using the communication box (Star-Oddi, Gardabaer, Iceland) and the associated Mercury 5.90 application software (Star-Oddi, Gardabaer, Iceland).

## Electronic supplementary material

Below is the link to the electronic supplementary material.

Supplementary Material 1

Supplementary Material 1

## Data Availability

All data generated referring to DEE and TWI measurement and data retrieved from data loggers and are presented in the paper. The datasets are also available from the corresponding author on request.
