# Supplementary figures and images for: Evidence for SARS-CoV-2 infected Golden Syrian hamsters (Mesocricetus auratus) reducing daily energy expenditure and body core temperature
Source: Sci Rep. 2024 Oct 6;14:23263. doi: 10.1038/s41598-024-73765-2 (PMC11456599; doi:10.1038/s41598-024-73765-2)

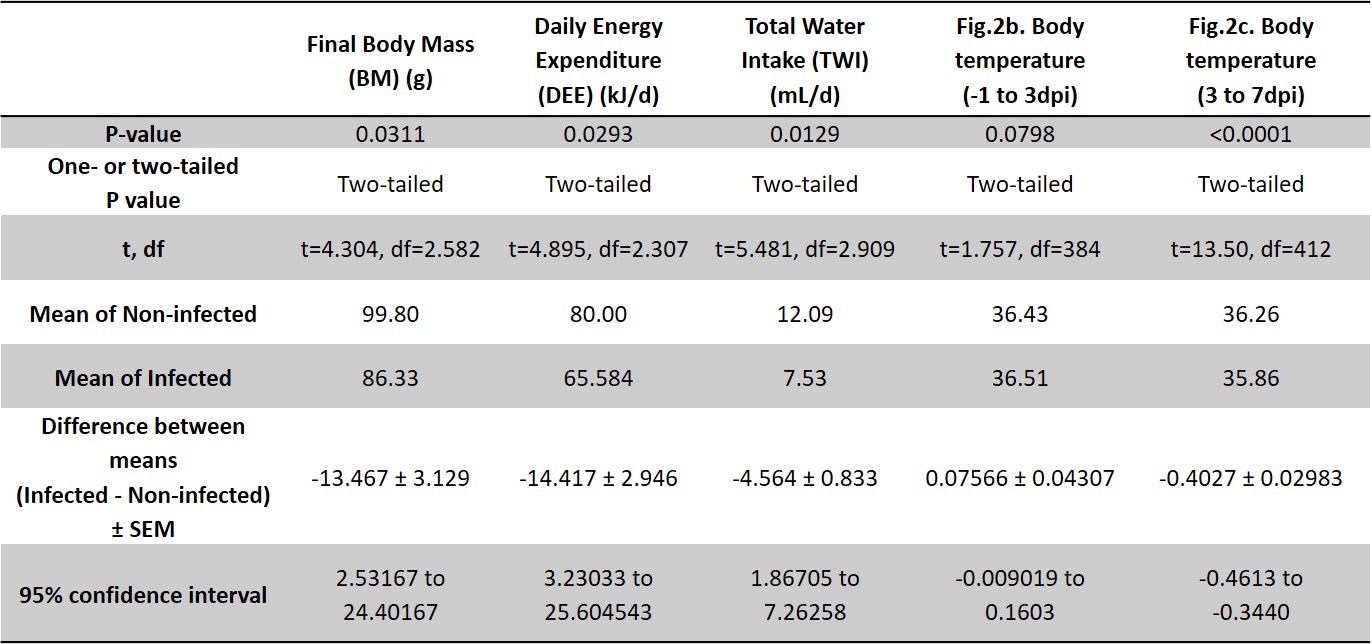

Supplement: Supplementary file 1 — Supplementary Material 1 [file 41598_2024_73765_MOESM1_ESM.jpg]
