# Supplementary material for: Evidence for SARS-CoV-2 infected Golden Syrian hamsters (Mesocricetus auratus) reducing daily energy expenditure and body core temperature
Source: Sci Rep. 2024 Oct 6;14:23263. doi: 10.1038/s41598-024-73765-2 (PMC11456599; doi:10.1038/s41598-024-73765-2)
Supplement: Supplementary file 2 — Supplementary Material 1 [file 41598_2024_73765_MOESM2_ESM.docx]

**Supplementary Table 1:** Statistical parameters of study for body mass (BM), daily energy expenditure (DEE), total water intake (TWI), and body temperature changes in non-infected and SARS-CoV-2 infected Golden Syrian Hamsters.
